# A rhoptry protein, localizing in the bulb region of rhoptries, could induce protective immunity against *Eimeria tenella* infection

**DOI:** 10.3389/fimmu.2023.1277955

**Published:** 2023-12-04

**Authors:** Tuan-yuan Shi, Si-han Zhou, Ya-ru Kong, Yuan Fu, Yan Liu, Wen-chao Yan, Yong-xue Zhou, Liang Zhang, Li-li Hao, Hong-chao Sun

**Affiliations:** ^1^ Institute of Animal Husbandry and Veterinary Medicine, Zhejiang Academy of Agricultural Sciences, Hangzhou, Zhejiang, China; ^2^ College of Animal Science and Technology, Henan University of Science and Technology, Luoyang, Henan, China; ^3^ Department of Epidemic Surveillance, Lingcheng Center for Disease Control and Prevention, Dezhou, Shandong, China; ^4^ Department of Animal Parasitology, College of Animal and Veterinaty Sciences, Southwest Minzu University, Chengdu, Sichuan, China

**Keywords:** *Eimeria tenella*, rhoptry protein, *Et*ROP21, localization, protective efficacy

## Abstract

**Background:**

Rhoptry organelle proteins (ROPs) secreted by apicomplexan parasites play important roles during parasites invasion and survival in host cells, and are potential vaccine candidates against apicomplexan diseases. *Eimeria tenella* (*E. tenella*) is one of the most noteworthy apicomplexan species, which causes hemorrhagic pathologies. Although dozens of putative *E. tenella* ROP sequences are annotated, most ROP proteins are not well studied.

**Methods:**

In this study, an *E. tenella* ROP21 gene was identified and the recombinant *Et*ROP21 protein (r*Et*ROP21) was expressed in *Escherichia coli*. The developmental expression levels, localization, and protective efficacy against *E. tenella* infection in chickens were studied.

**Results:**

An *Et*ROP21 gene fragment with an open reading frame (ORF) of 981 bp was obtained from the Beijing strain of E. tenella. The r*Et*ROP21 has a molecular weight of approximately 50 kDa and was recognized by r*Et*ROP21-immunized mouse serum. Two specific protein bands, about 43 KDa and 95 KDa in size, were detected in the whole sporozoite proteins using the r*Et*ROP21-immunized chicken serum. RT-qPCR analysis of the *E. tenella* ROP21 gene (*Et*ROP21) revealed that its mRNA levels were higher in merozoites and sporozoites than in sporulated and unsporulated oocysts. Immunofluorescence and immunoelectron analyses showed that the *Et*ROP21 protein predominantly localizes in the bulb region of rhoptries distributed at anterior, posterior, and perinuclear regions of *E. tenella* sporozoites. Immunization and challenge experiments revealed that immunizing chickens with r*Et*ROP21 significantly increased their average body weight gain while decreasing mean lesion score and oocyst output (*P* <0.05). When compared with the challenged control group, the r*Et*ROP21-immunized group was associated with a significantly higher relative weight gain (90.2%) and a greater reduction in oocyst output (67%) (*P* <0.05). The anticoccidial index of the r*Et*ROP21-immunized group was 163.2. Chicken serum ELISA revealed that the levels of the specific anti- r*Et*ROP21 antibody, IFN-γ, and IL-4 were significantly higher in the r*Et*ROP21-immunized group than in the challenged control group (*P* <0.05).

**Conclusion:**

These results indicate that r*Et*ROP21 can induce a high level of specific immune response and it is a potential candidate for the development of vaccines against *E. tenella* infection in chickens.

## 1 Introduction

*Eimeria tenella* is the causative agent of chicken coccidiosis, which causes an estimated annual loss of ~ £10.4 billion annually worldwide (1). Traditional measures against chicken coccidiosis include the use of anticoccidial drugs and live oocyst vaccines (2). However, the effectiveness of these measures has been limited by the emergence of drug-resistant strains, growing consumer concerns about food safety, virulence reversion, and production challenges. Therefore, novel and cost-effective anticoccidial control measures are needed (3, 4). The development of effective coccidiosis vaccines require the identification of highly protective antigens (5). However, because *E. tenella* expresses about 6,700 proteins across its life cycle (6), it is necessary to first screen the *E. tenella* proteins and identify the protective antigens among so many parasite proteins. Apicomplexan parasites, including *Eimeria*, *Toxoplasma gondii*, and *Plasmodium*, have unique apical secretory organelles called rhoptries, dense granules, and micronemes, which are necessary for cell invasion, survival, and parasite growth (7, 8). Rhoptries are an arsenal of secreted virulence factors involved in apicomplexan pathogenicity (9). Hence, rhoptry organelle proteins (ROPs) are considered promising vaccine candidates against apicomplexan diseases (10). Indeed, several *T. gondii* ROPs, including *Tg*ROP1, 5, 7, and 17, have been reported to effectively stimulate protective immunity and to significantly reduce brain cyst loads in immunized animals (11–14). Several *E. tenella* ROPs, including *Et*rop5405, *Et*rop5905 (EtROP35), *Et*rop27705 (EtROP30), and *Et*rop5190 (*Et*ROP17) have been also reported to effectively stimulate protective immunity and reduce oocyst output in immunized chickens (15–18). In this study, we identified *Et*ROP21, an *E. tenella* ROP protein, and investigated its localization in sporozoites, as well as its protective efficacy against *E. tenella* infection. Our results provide a foundation for the development of effective vaccines against chicken coccidiosis.

## 2 Materials and methods

### 2.1 Animals and ethics

Coccidia-free chickens (one-day-old) were obtained from a local poultry breeding center (Xiaoshan, Hangzhou, China). These chickens were then raised at the experimental animal center of Zhejiang Academy of Agricultural Sciences in coccidia-free conditions and were given free access to forage (without anticoccidial drugs) and cold boiled water. All animal studies and protocols were approved by the institutional animal care committee of the Zhejiang Academy of Agricultural Sciences (approval No. 2022ZAASLA001).

### 2.2 *E. tenella* propagation, isolation, and purification

Two-week-old specific pathogen free (SPF) chickens were used to propagate oocysts of the *E. tenella* Beijing strain, which was obtained from Professor Xun Suo’s laboratory at China Agricultural University. Briefly, 1×10^4^ sporulated oocysts of the Beijing strain were orally inoculated to the chickens. Seven to nine days after the inoculation, unsporulated oocysts were collected and purified according to a modified method (19). The gained oocysts were sporulated in 2.5% potassium dichromate solution in a constant-temperature shaker (Shanghai Zhichu Instrument, China) at 28°C with a rotation speed of 200 rpm. In order to purify sporozoites from the oocysts, a tissue-preparation instrument (Gering, China) was used to release sporocysts. Then, sporozoites were excysted using a modified excystation buffer made of phosphate-buffered saline (1×PBS, pH 7.4), 0.75% trypsin, and 10% chicken bile extract, and purified using DE-52 anion-exchange chromatography (20). Merozoites were isolated and purified according to a modified method (21) as follows: ceca were taken from the chickens at 120 h post the inoculation of *E. tenella* sporulated oocysts and washed with the sterilized 1×PBS. And then mucosal tissues were scraped from the ceca and harvested with a digestion solution containing 1 mg/mL hyaluronidase, 120 mmol/L NaCl, 20 mmol/L Tris-HCl (pH7.4), 3 mmol/L K_2_HPO_4_, 1 mmol/L CaCl_2_, and 1 mg/mL bovine serum albumin (BSA) at 37°C for 60 min. The digestive mixture containing merozoites was filtrated with 200-mesh sieves and centrifuged to gain merozoite precipitation. The merozoites were further purified using erythrocyte lysis buffer (Solarbio, China), and Percoll density gradient centrifugation at 4,000 rpm for 5 min. The finally purified merozoites were stored at 4°C for use. All oocysts, sporocysts, sporozoites, and merozoites were enriched using horizontal centrifugation (Cence, China).

### 2.3 Amplification of the *Et*ROP21 gene and bioinformatics analysis

*E. tenella* sporozoites (5×10^7^) RNA was extracted using Trizol reagent (Invitrogen, USA), and cDNA was prepared using a *TransScript* ^®^ First-Strand cDNA Synthesis SuperMix kit (TransGen, China). The gene sequence of *Et*ROP21 was amplified by polymerase chain reaction (PCR). Primers for PCR amplification of the ROP21 gene were designed according to the nucleotide sequence (NCBI accession No. XM_013377531) of *E. tenella* (Houghton strain); 12 specific primers (11 upstream and one downstream) were synthesized, and the actin gene was used as control. All of the primers are listed in **Table 1**. The PCR products were identified using 1% agarose gel and purified fragments were cloned into the pMD19-T vector. Then, the pMD19-T- *Et*ROP21 plasmid was transformed into trans-t1 *E. coli* cells (TransGen, China). After cultivation on Luria broth (LB) agar plates at 37°C for 12 h, the clones were picked and identified by PCR, and the positive plasmid of pMD19-T- *Et*ROP21 was further verified via sequencing. Finally, the obtained sequences were blasted in NCBI, and a phylogenetic tree was constructed using the MEGA 6 software (all of the reference sequences are listed in **Table 2**). In order to predict the molecular weight and isoelectric point of ROP21 protein gained in the study, the ExPASy ProtParam tool was applied. The SignalP 4.1 server was utilized for prediction of the signal peptide. In addition, ROPs are usually considered promising vaccine antigens; in order to analyze the antigenicity of the ROP21 protein, the DNAMAN (9.0) tool was utilized for prediction of the antigenic epitopes.

**Table 1:** Primers designed to amplify the ROP21 gene sequence of *Eimeria tenella*.

Number	Primer name	Primer sequence
1	rop21-F1	5’-ATGGGGGCCCCCAGCCCTGC
2	rop21-F2	5’-GTCTTAACTGCTGCGCTGCTGG-3’
3	rop21-F3	5’-ATGAGGACTTGGGGCCCAGTCT-3’
4	rop21-F4	5’-ATGTCTTTGGCGGACCGC-3’
5	rop21-F5	5’-CCCTGGACGACAGCACCT-3’
6	rop21-F6	5’-GGGGGGGCCCCCGAGGAGGGGGGG GCCCCC-3’
7	rop21-F7	5’-ATGAGAAGTACCCTCGGCTTGG-3’
8	rop21-F8	5’-ATGGGGCCCCAGCTGCAGCAGCAC-3’
9	rop21-F9	5’-GCAGAGAGCAAAAGGGCCCCCCAG-3’
10	rop21-F10	5’-GACCCGCGGGTGCTGCAGCCG-3’
11	rop21-F11	5’-ATGCGAATAATGTTTTTGGACG-3’
12	rop21-R	5’-GGTTTGTGGGAGAAAAGAGGC-3’
13	actin-F	5’-GTGCGAAAATGGCGGACGAAGAGGT-3’
14	actin-R	5’-TTCACGACGGCAGGGTCCAACGCAT-3’

**Table 2:** GenBank reference sequences used in construction of the phylogenetic tree.

Species	Source/Location	GenBank accession no
*Eimeria acervulina*	Houghton strain from chicken, United kingdom	XM_013393718.1
*Eimeria maxima*	Houghton strain from chicken, United kingdom	XM_013479882.1
*Eimeria necatrix*	Houghton strain from chicken, United kingdom	XM_013584203.1
*Toxoplasma gondii*	/	XM_018781319.1
*Besnoitia besnoiti*	Cattle, Germany	XM_029358848.1

”/” indicates no information.

### 2.4 Analysis of *Et*ROP21 expression at various *E. tenella* developmental stages

*Et*ROP21 mRNA levels in the developmental stages of sporozoites, second-generation merozoites, sporulated oocysts, and unsporulated oocysts were determined using RT-qPCR as previously described (22). Briefly, *E. tenella* total RNA was extracted at these four stages and reverse transcribed as described in section 2.3. *Et*ROP21 transcript levels were then determined on a 7500 real-time PCR system (ThermoFisher, USA) using the primers shown in **Table 1** and actin as the reference gene. Relative gene expression was determined using the 2^-ΔΔCt^ method (23). The data represent three independent experiments.

### 2.5 Expression and preparation of recombinant *Et*ROP21 protein

The *Et*ROP21 gene fragment was cloned in pMD19-T vector, and it was then double-digested by *Kpn* I/*Xho* I restriction enzymes. The purified *Et*ROP21 fragment was ligated into the pET32a expression vector leading to a recombinant plasmid, pET32a-*Et*ROP21. Then, the recombinant plasmid was transfected into competent *E. coli* BL21 (DE3) cells (TransGen, China), and the bacteria were grown in LB plates containing 100 μg/ml ampicillin. After an overnight incubation at 37°C, the clones were picked and confirmed with PCR, double digestion with restriction enzymes, and gene sequencing. The confirmed positive clone was inoculated into LB medium with ampicillin and cultured at 37°C with shaking at 200 rpm until it reached an optical density (OD600) of 0.6-0.8. The r*Et*ROP21 protein was induced with 1 m M IPTG and shaking at 37°C with 200 rpm for 4 h, and the precipitate of the bacteria expressing r*Et*ROP21 protein was collected after being centrifuged at 8,000 rpm for 5 min at 4°C. The precipitate was re-suspended in PBS and sonicated at 40 W for 30 min (Ningbo Scientz, China), and then the recombinant protein was identified by SDS-PAGE. The r*Et*ROP21 protein was further purified using Ni2+-NTA (Thermo Scientific, USA). Finally, the concentration of the protein was measured using a BCA protein assay kit (Beyotime, China), and the obtained protein was stored at -80°C until use.

### 2.6 Western blot analysis

In order to prepare sporozoite proteins, 5×10^7^ *E. tenella* sporozoites were treated with RIPA lysis buffer (Solarbio, China) containing 1 mM protease inhibitor phenylmethanesulfonyl fluoride (PMSF) and sonicated on ice for 10 min. The treated sporozoite mixture was centrifuged at 12,000 rpm for 10 min at 4°C to gain supernatant. The r*Et*ROP21 recombinant protein and the sporozoite proteins were separated using 12% SDS-PAGE gels, and then were transferred onto nitrocellulose (NC) filter membrane at 200 mA for 15 min using a Bio-Rad transfer system (Bio-Rad, USA); bands of the transferred NC membrane were blocked with 5% skim milk at 4°C overnight. Sera (1:250 dilution with PBS) from r*Et*ROP21-immunized or unimmunized mice or chicken were added and incubated at 37°C for 2 h. The bands were then incubated with horseradish peroxidase (HRP)-conjugated goat anti-mouse IgG (1:6000 dilution with PBS, Beyotime, China) for 2 h at 37°C after being washed with PBST three times. Finally, an enhanced chemiluminescence kit (Sangon Biotech, China) was used to visualize the bands. Six-week-old SPF mice were used to produce polyclonal antibodies for r*Et*ROP21. Briefly, 100 μg of purified r*Et*ROP21 formulated with Freund’s complete adjuvant (Thermo Fisher Scientific, USA) (1:1) were subcutaneously injected into mice. The second and third booster immunization were performed at 14-day intervals, with 50 ug of r*Et*ROP21 protein plus Freund’s incomplete adjuvant. Seven days after the third immunization, mouse sera were separated and stored at -80°C for use.

### 2.7 Immunofluorescence assay and immunoelectron assay

*Et*ROP21 localization was observed using indirect immunofluorescence assay (IFA) and immunoelectron assay (IEA). The IFA protocol was as follows: purified sporozoites (1×10^7^) were fixed with 2% paraformaldehyde for 20 min and centrifuged at 4,000 rpm for 5 min. The sporozoites were permeabilized with 0.2% Triton-X-100 for 10 min at 37°C and then were blocked with 5% BSA after being washed three times with 0.01 M PBS. Primary anti-r*Et*ROP21 antibody (mouse) diluted 1/100 with PBS was incubated for 1 h at room temperature. After washes with PBS, the sporozoites were incubated with secondary antibody Alexa Fluor^®^ labeled goat anti-mouse 488 (Beyotime, China, 1/500 dilution). DAPI was used to stain the nuclei following further washes, and the sample was examined by fluorescent microscope (SOPTOP, China). For IEA, 1×10^8^ freshly purified sporozoites were fixed in 3% paraformaldehyde (Shanghai Macklin Biochemical Ltd, China) + 2% glutaraldehyde (ThermoFisher Scientific Ltd, USA) in 0.2 M phosphate buffer (PB), pH 7.2, at 4°C overnight. They were then washed thrice using PB and centrifugation at 4,000 rpm for 5 min at 4°C. The sporozoites were then dehydrated with 30% ethanol for 30 min at 4°C, and then with 50%, 70%, 80%, 90%, 95%, and 100% ethanol for 60 min at −20°C. They were then infiltrated with 1:1 and 2:1 LR gold resin/ethanol at −20°C for 2 h, with 100% LR gold resin (Agar Scientific Ltd, UK) overnight, at −20°C, and then with 0.06% and 0.12% benzil (London Resin company, UK) in LR gold resin at −20°C for 6 h and overnight, respectively. The sporozoites were centrifuged at 4,000 rpm for 5 min at 4°C following each step of dehydration or infiltration. The supernatants were discarded. Samples were then embedded in 0.12% benzil in LR gold resin with ultraviolet radiation at −20°C for 96 h. They were then trimmed, frozen in liquid nitrogen, and sectioned using an ultra-thin microtome (Leica UC6, Germany). The nanometer sections were then blocked with 1% BSA and 0.02% PEG2000 in PBS (pH 7.0) for 30 min at room temperature and incubated with serum from r*Et*ROP21-immunized mice for 1 h at room temperature, followed by incubation with rabbit-anti-mouse IgG secondary antibody conjugated with 15-nm gold particles (Abcam, UK) for 1 h at room temperature. After each reaction, the sections were washed thrice with PBS and imaged using a transmission electron microscope (Hitachi 7650, Japan).

### 2.8 Analysis of immuno-protection induced by r*Et*ROP21

Sixty (one-week-old) coccidia-free chickens were randomly divided into three groups of 20 chickens with similar mean body weights per group (**Table 3**). Immunization procedures for the experimental chickens were as follows: the r*Et*ROP21 protein with 100 μg/chicken was used for chickens of the *Et*ROP21 group. PBS was used for chickens of the challenged and unchallenged control groups. The r*Et*ROP21 protein and PBS were emulsified in complete Freund’s adjuvant before use and injected subcutaneously into the neck of the chickens. Two hundred µg of r*Et*ROP21 and PBS emulsified in Freund’s incomplete adjuvant were used in the second and third immunizations with 14-day intervals. Blood samples were collected from all the experimental groups on the seventh day after the third immunization and sera were isolated for use in antibody and cytokine (IFN-γ, IL-2, IL-4, TGF-β) detection. Chickens in the r*Et*ROP21-immunized and challenged control group were orally challenged with 2×10^4^ sporulated *E. tenella* oocysts and chickens in the unchallenged control group were orally administered the same volumes of PBS. In order calculate *E. tenella* oocysts, chicken droppings from each experimental group were collected separately on the sixth to ninth day post the challenge. All the chickens were weighed and slaughtered on the ninth day after the challenge. The cecum and its content from chickens of each group was collected separately. The McMaster counting method was used to count *E. tenella* oocysts in the chicken droppings and the cecal contents. The oocyst decrease ratio was calculated using the formula: (total number of oocysts from the challenge control group-total number of oocysts from r*Et*ROP21-immunized group)/total number of oocysts from the challenge control group × 100%. Body weight gain was calculated as the following: mean body weight per group on the slaughter day-mean body weight per group on the challenge day. Cecal lesion scores were determined on a graded scale from 0 (normal) to 4 (severe), as previously described by Johnson and Reid (24). The anticoccidial index (ACI) is a synthetic criterion for determining anticoccidial effects and was calculated using the formula: (relative rate of weight gain + survival rate) × (lesion value + oocyst value). ACIs of >180, 160 < ACI ≤ 179, 120 < ACI ≤ 159, and <120 were considered as an excellent anticoccidial effect, good anticoccidial effect, generally effective anticoccidial effect, and invalid, respectively (25).

**Table 3:** Groups of the experimental animal and immunization program.

Groups	Number	Immune dosage (total volume 0.2 mL per chicken)	Challenge dosage (sporulated oocysts per chicken)
unchallenged control	20	PBS+Freund’s complete adjuvant	0
challenged control	20	PBS+Freund’s incomplete adjuvant	2×10^4^
rEtROP21 immunized	20	100 µg/200 µg rEtROP21+Freund’s incomplete adjuvant	2×10^4^

100 µg rEtROP21 was used for the first immunization and 200 µg rEtROP21 was used for the second and third immunizations.

### 2.9 Antibody and cytokines assays

The level of the specific anti-*Et*ROP21 antibody was determined using enzyme-linked immunosorbent assay (ELISA). Briefly, r*Et*ROP21 protein was diluted to 1 μg/mL with 50 mM carbonate buffer (PH 9.6) and the ELISA plate was coated with 100 μg/well of r*Et*ROP21 protein at 4°C overnight. The ELISA plate was blocked for 2 h using 100 μL of 5% skim milk and washed with PBST three times. Diluted sera (a gradient of 1:100 to 1:12,800) were added in triplicate and incubated for 2 h at room temperature. HRP-conjugated goat anti-chicken IgG secondary antibody (Beyotime, China, 1/10,000 dilution) was added after three washes, and the plate was incubated for 1 h at 37°C. After the final washes, a tetramethylbenzidine (TMB) substrate solution was added into the wells followed by incubation at 37°C for 20 min and subsequently the reaction was stopped after adding 50 μL of stop solution (2M H_2_SO_4_). The absorbance was read at 450 nm. Cytokine levels, IFN-γ, IL-2, IL-4, and TGF-β in the chicken sera from each group were detected using ELISA kits (Cusabio, Wuhan Huamei Biotech, China). The assay was performed in three independent experiments.

### 2.10 Statistical analysis

Statistical analyses were performed using GraphPad Prism version 8.0 (GraphPad Prism, San Diego, CA). The differences in cytokines production, antibody level, mean body weight gain, oocyst output per chicken, and mean lesion scores were determined with a one-way analysis of variance (ANOVA). All of the experiments were done in triplicate and three independent experiments and the results are shown as the arithmetic mean ± standard deviation. *P <*0.05 indicates statistically significant differences.

## 3 Results

### 3.1 Determination of *Et*ROP21 transcript levels and construction of a recombinant *Et*ROP21 expression plasmid

**Figure 1:**
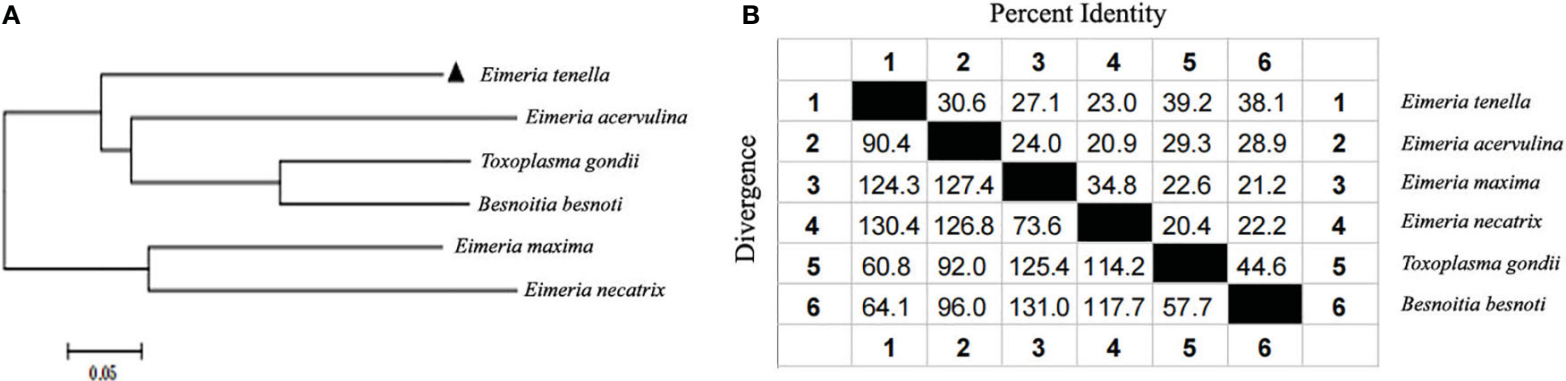
Phylogenetic tree construction and homology analysis based on ROP21 genes. **(A)** A phylogenetic tree based on ROP21 gene sequences from different apicomplexan species. **(B)** Similarity and differences between the ROP21 gene sequences of various apicomplexan species.

**Figure 2:**
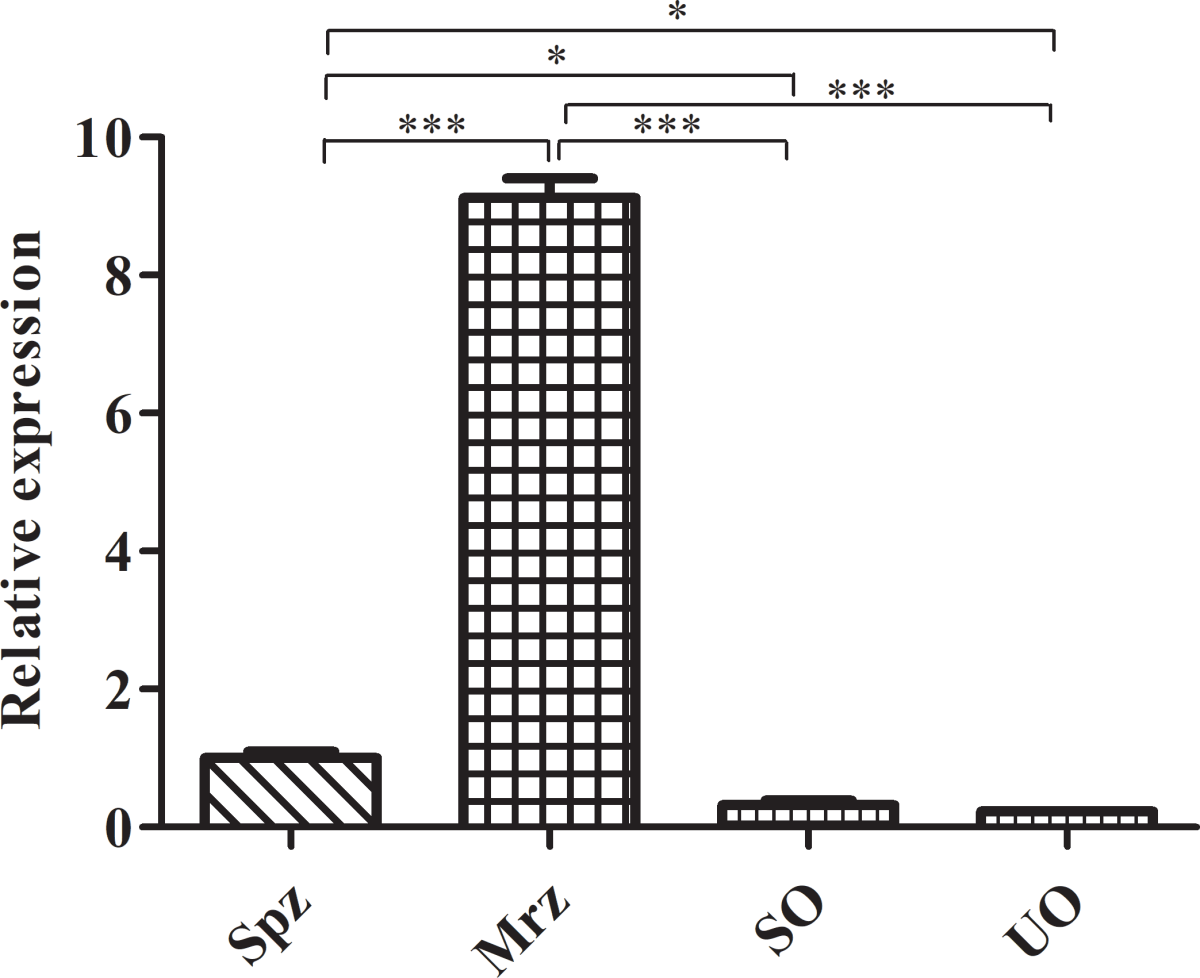
RT-qPCR analysis of ROP21 mRNA levels at different *E. tenella* developmental stages. UO, unsporulated oocysts; SO, sporulated oocysts; Spz, sporozoites; Mrz, merozoites. * and *** indicate *P <*0.05 and <0.001, respectively.

Of the 12 specific primers used to amplify *Et*ROP21 gene fragments PCR, only one primer pair (rop21-F11 and rop21-R) generated a single, 981-bp DNA band from the *E. tenella* Beijing strain (**Figure S1A**). Actin, which was used as the reference gene, was amplified correctly. Sequence analysis indicated that the *Et*ROP21 gene fragment amplified from the Beijing strain was 100% identical to the partial sequence of a putative *E. tenella* ROP21 gene from the Houghton strain (Gene ID: 25252056). A phylogenetic tree constructed using four ROP21 gene sequences from other apicomplexan parasites (**Table 2**) indicated that they share a lower percent identity with the sequence from *E. tenella* (**Figure 1**). Bioinformatics analysis revealed that the 981-bp *Et*ROP21 gene fragment encoded a protein with a predicted molecular weight of 36.89 kDa. The isoelectric point of *Et*ROP21 protein was 8.69, and eight potential antigenic epitopes were predicted. There were no signal peptide regions in the *Et*ROP21 protein (**Table 4**). A recombinant *Et*ROP21 expression plasmid (pET32a (+)-*Et*ROP21) was successfully constructed (**Figure S1B**). RT-qPCR analysis of RNA from sporozoites, second-generation merozoites, and sporulated and unsporulated oocysts revealed that the *Et*ROP21 mRNA levels were markedly high in second-generation merozoites and sporozoites than in sporulated and unsporulated oocysts (**Figure 2**).

**Table 4:** The predicted antigenic epitopes of EtROP21.

Number	Sequence	Position	Score value
1	WAGVHCKAD	143-151	1.163
2	GGGVVSAVDS	8-17	1.158
3	YSWVVDVGDS	65-74	1.154
4	SWKYVADVWSSVVMD	43-57	1.133
5	RRAVKVCRR	20-28	1.131
6	MVRVAKVMYA	81-90	1.120
7	VRAAAS	195-200	1.075
8	SDKANVH	100-106	1.033

### 3.2 Expression and purification of recombinant *Et*ROP21 protein

**Figure 3:**
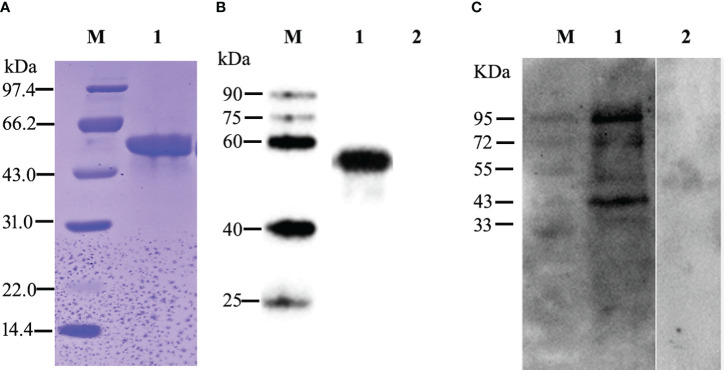
Expression, purification, and Western blot analysis of the r*Et*ROP21 protein. M: protein marker. **(A)** SDS-PAGE analysis of the purified r*Et*ROP21 protein. Lane 1: the r*Et*ROP21 protein was purified using nickel ion affinity chromatography. **(B)** Western blot analysis of the recombinant *Et*ROP21 protein. Lanes 1 and 2: recombinant *Et*ROP21 reacted with immunized and unimmunized mouse sera, respectively. **(C)** Western blot analysis of the *Et*ROP21 protein in *E. tenella*. Lanes 1 and 2: total proteins from *E. tenella* sporozoites reacted with immunized and unimmunized chicken sera, respectively.

The recombinant target *Et*ROP21 protein (r*Et*ROP21) was induced in *E. coli* strain BL21 (DE3) by the addition of IPTG to a final concentration of 1 mM/L and incubation at 37°C and 200 rpm for 4 h. The r*Et*ROP21 had a molecular weight of approximately 50 kDa, which included the Trx and His-tag protein expressed from the pET32a (+) vector. The recombinant rEtROP21 protein appeared as inclusion bodies. Therefore, the actual size of r*Et*ROP21 (50 kDa) in the SDS-PAGE was in agreement with the theoretical size. Purified r*Et*ROP21 was isolated using nickel ion affinity chromatography and recovery from SDS-PAGE (**Figure 3A**). Western blot analysis revealed the presence of a specific anti-*Et*ROP21 antibody in sera from r*Et*ROP21-immunized mice or chicken, and that the antibody reacted with both the recombinant protein and whole sporozoite proteins. The band size of the *Et*ROP21 protein in the Western blot was consistent with its theoretical size and sera from unimmunized mice or chicken did not react with r*Et*ROP21 or with whole sporozoite proteins (**Figures 3B, C**). In addition, there was another larger protein band, about 95 KDa in size, seen in the whole parasite protein. We speculate that the larger protein may be the dimer form of the *Et*ROP21 protein.

### 3.3 *Et*ROP21 localization in *E. tenella* sporozoites

**Figure 4:**
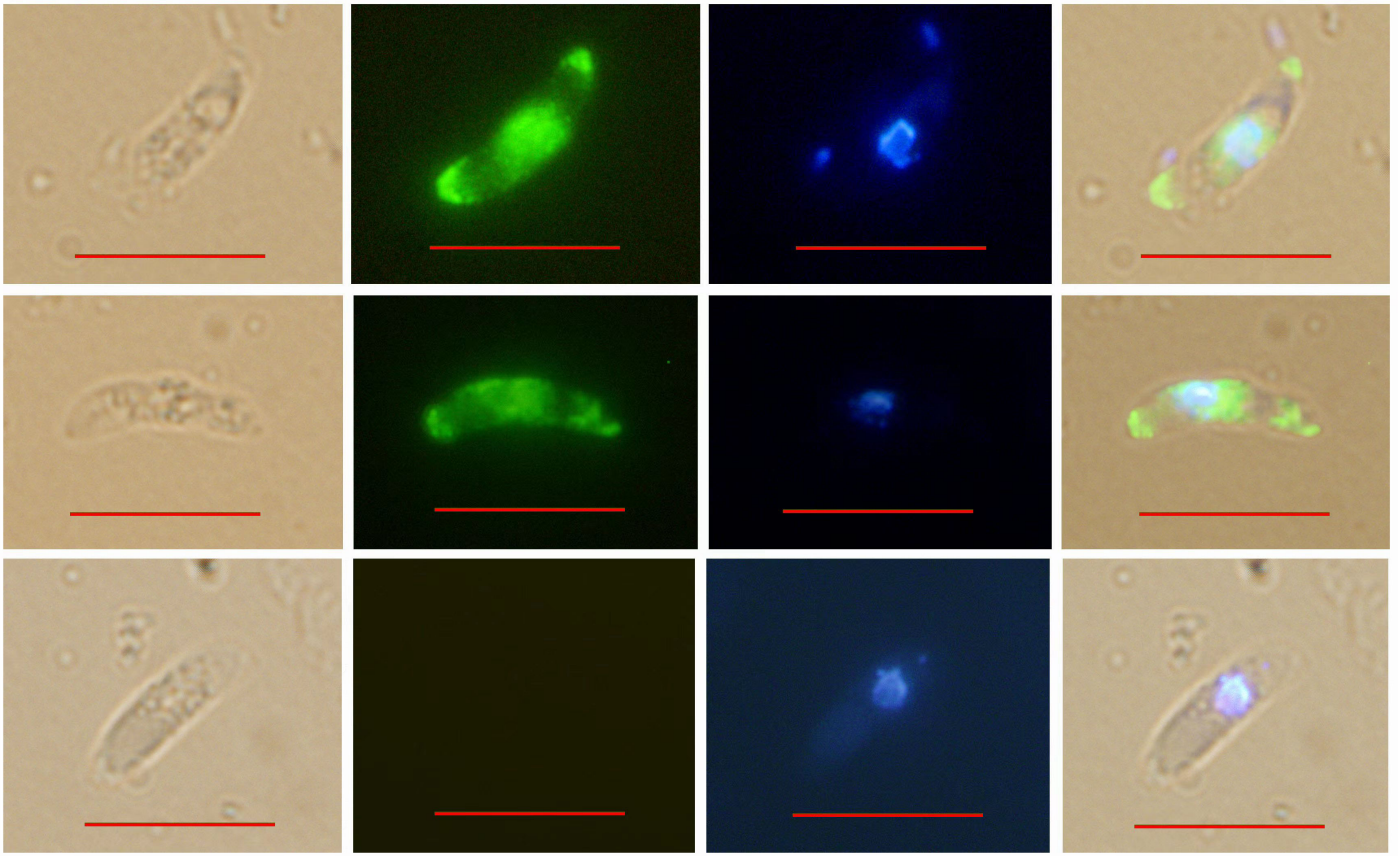
Determination of *Et*ROP21 localization in *E. tenella* sporozoites using indirect immunofluoresence. BRI: sporozoites under bright field imaging. FLU: sporozoites stained with Alexa Fluor 488-labeled goat anti-mouse IgG under green fluorescence imaging. UV: sporozoites stained with DAPI under UV imaging. Mer: merged images. Scale bar: 10 μm.

**Figure 5:**
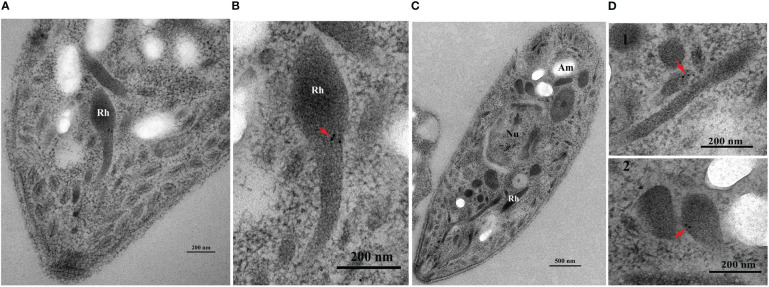
Determination of *Et*ROP21 localization in *E. tenella* sporozoites’ rhoptries using immunoelectron analysis. **(A, B)** The anterior region **(A)** and rhoptry **(B)** of a sporozoite stained with gold particles. **(C, D)** A whole sporozoite **(C)** and its rhoptries **(D)**. (D1–D2) Rhoptries localization at the anterior (D1) and posterior (D2) regions of a sporozoite stained with gold particles. “Rh”, “Nu”, and “Am” represents “Rhoptries”, “Nucleus”, and “Amylopectin granules”, respectively.

IFA indicated that *Et*ROP21 was mainly distributed at the anterior, posterior, and perinuclear regions of *E. tenella* sporozoites but not in the refractive bodies (**Figure 4**). IEA further confirmed that *Et*ROP21 was mainly localized in the bulb region of the rhoptries (**Figure 5**).

### 3.4 r*Et*ROP21 exhibits immunoprotection against *E. tenella* infection

**Figure 6:**
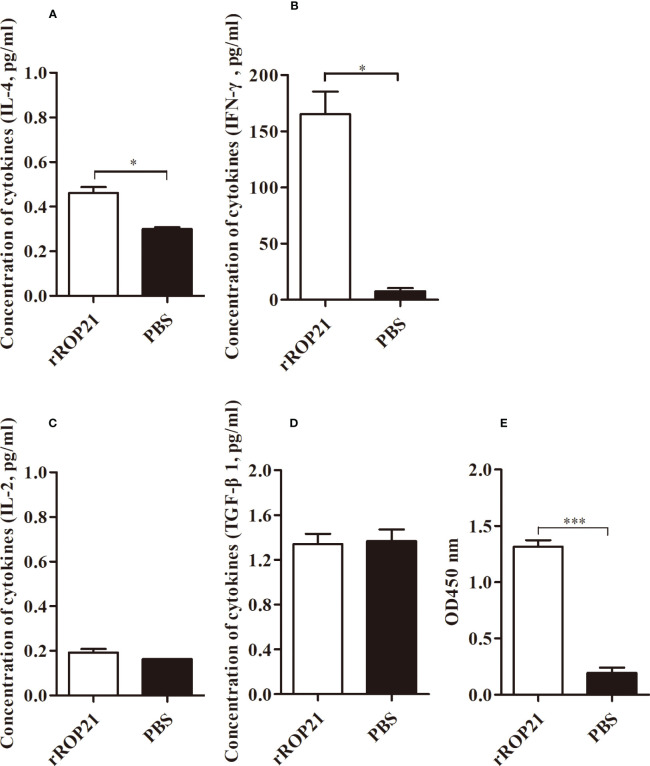
ELISA analyses of the levels of IL-4, IFN-γ, IL-2, TGF-β, and a specific anti-r*Et*ROP21 antibody in chicken sera (n=4). **(A–D)** Levels of IL-4, IFN-γ, IL-2, and TGF-β in the r*Et*ROP21 and PBS-immunized groups, respectively. **(E)** Levels of the anti-r*Et*ROP21 specific antibody in the r*Et*ROP21 and the PBS-immunized control group, respectively. * and *** indicate *P <*0.05 and *P <*0.001, respectively.

No deaths were observed in all experimental groups (the r*Et*ROP21-immunized group and PBS- immunized group). Whereas the challenged control group exhibited severe clinical symptoms, including bloody droppings, decreased appetite, and depression, the r*Et*ROP21-immunized group showed only mild symptoms on the fifth day after the challenge. The average weight gain was significantly higher in the r*Et*ROP21-immunized group (443.3 ± 47.30 g) than in the challenged control group (*P <*0.05), which had a relative weight gain of 90.23%. The average oocyst output was 8.98×10^7^ ± 9.21×10^6^ per chicken in the r*Et*ROP21-immunized group and 2.76×10^8^ ± 3.58×10^7^ in the challenged control group. No oocysts were observed in the unchallenged control group. Compared with the challenged control group, the r*Et*ROP21-immunized group had significantly lower oocyst excretion (67.42% oocyst reduction, *P <*0.05). The cecal lesion score of the r*Et*ROP21-immunized group was lower than that of the challenged control group. No lesions were observed in the unchallenged controls. The ACI value of the r*Et*ROP21-immunized group was 163.23 (**Table 5**). According to the judgment criteria of ACI in the Materials and Methods section, r*Et*ROP21 showed a good anticoccidial effect. The ELISA results revealed that levels of specific anti-*Et*ROP21 antibody, IFN-γ, and IL-4 in chicken sera were significantly higher in the r*Et*ROP21-immunized group than in the unimmunized control group (*P <*0.05, **Figures 6A–E**). Because IFN-γ and IL-4 are mainly associated with host humoral and cellular immune responses, this indicates that the r*Et*ROP21 protein stimulated the chickens’ humoral and cellular immune responses.

**Table 5:** Protective efficacy of rEtROP21 against *Eimeria tenella* infection.

Groups	Mean body weight gain (g)	Relative body weight gain rate (%)	Oocyst output per chicken (×10^4^)	Reduced percentage of oocyst excretion (%)	Survival rate (%)	Mean lesion scores	ACI
unchallenged control	491.3 ± 42.72_a_	100	0	100	100	0	200
challenged control	401.8 ± 52.45_b_	81.78	8.98×10^7^ ± 9.21×10^6^_b_	0	100	3.8 ± 0.42_a_	133.78
rEtROP21 immunized	444.3 ± 42.97_c_	90.23	2.76×10^8^ ± 3.58×10^7^_a_	67.42	100	2.6 ± 0.52_a_	163.23

_a_, _b_, _c_; The same subscripts in a column of data indicate that the difference is not significant.

## 4 Discussion

This study identified a rhoptry protein, *Et*ROP21, from the *E*. *tenella* Beijing strain. Based on RT-qPCR analysis, we estimated that the *Et*ROP21 gene fragment transcribed in the *E. tenella* sporozoite stage was 981-bp long, which was markedly shorter than the putative whole *Et*ROP21 gene from the *E. tenella* Houghton strain (Gene ID: 25252056), probably because only a partial sequence of the *Et*ROP21 gene was expressed by *E. tenella* sporozoites. RT-qPCR analysis showed that the expression levels of *Et*ROP21 were higher in the extracellular sporozoite and second-generation merozoite stages (26), which is consistent with our observation. Rhoptry organelle proteins, one type of specific secretory proteins of apicomplexa, are usually considered as having a signal peptide in the N-terminal region. However, no signal peptide was predicted in the *Et*ROP21 protein identified in this study. The reasons could be that the *Et*ROP21 protein identified in this study is a truncated version of a larger protein, lacking its signal peptide. In addition, considering the two specific protein bands detected in the whole sporozoite proteins with the Western bot, in which the smaller protein about 43 KDa was similar in molecular size with the theoretical molecular weight calculated from the 981-bp *Et*ROP21 gene fragment amplified in the study, the other main possibility was that the *Et*ROP21 protein could be another type of ROP without the signal peptide. Similar findings have been reported in *T. gondii* ROPs, in which *T. gondii* ROP30 was also predicted containing no signal peptide in the N-terminal region (27). We speculate that the larger protein, about 95 Kda, observed in the whole sporozoite protein may be the dimer form of the *Et*ROP21 protein. This was in agreement with the study in *T. gondii*, in which two forms of *Tg*ROP21 also occurs, with the predicted molecular weights of 86 kDa and 51.4 kDa, and the smaller form was highly expressed both at tachyzoite and bradyzoite stages, but the larger form was poorly expressed at the bradyzoite stage (27).

Using IFA, we found that in *E. tenella* sporozoites, the *Et*ROP21 protein localizes at two terminal and perinuclear regions. This is consistent with the findings by Burrell et al., that although rhoptries predominantly localize at the apical end of sporozoites, they are also present at the central and posterior parts of the cell (28). This *Et*ROP21 immunolocalization in *E. tenella* sporozoites was consistent with previous reports on the immunolocalization of *Et*ROP30 and *Et*ROP35 (16, 18). Rhoptries are club-shaped organelles that contain a rounded bulb region and an elongated neck region. Our immunoelectron analysis showed that the *Et*ROP21 protein mainly localized in the bulb region of rhoptries, which is different from the localization of the *T. gondii* ROP21 protein. *Tg*ROP21 is primarily secreted in constitutive secretory vesicles into parasitophorous vacuoles (PV) rather than localizing in rhoptries in storage form (27).

Both host humoral and cellular immunity defend against pathogen infection. In this study, we found that r*Et*ROP21-immunized chickens have high levels of a specific anti-r*Et*ROP21 IgG. Higher levels of IFN-γ and IL-2 (Th1-like cytokines) and IL-4 (Th2-like cytokines) were also detected in the r*Et*ROP21-immunized group, which showed a mixed Th1/Th2- immune response. Because IFN-γ plays an important role in the immunoregulation of avian coccidia infection (29), its release has been used to screen for effective protective antigens against *E. tenella* infection (30). IL-4 also enhances IFN-γ production during the chronic stage of *T. gondii* infection (31) and is prominently involved in counterbalancing immune responses by downregulating IFN-γ secretion (32). In the current study, chickens vaccinated using the r*Et*ROP21 protein exhibited high levels of IFN-γ and IL-4, indicating that r*Et*ROP21 could protect chicken from *E. tenella* infection by inducing humoral and cellular immune responses.

In addition, animal protection experiments revealed that r*Et*ROP21 robustly protects against *E. tenella* infection, suppressing oocyst levels by up to 67.42%. Such a high level of protection has also been reported using a DNA vaccine based on *T. gondii*’s ROP21, which significantly prolonged survival time and decreased the number of brain cysts (33), as well as other *E. tenella* rhoptry proteins, including Etrop5405, Etrop5905 (EtROP35), Etrop27705 (EtROP30), and Etrop5190 (EtROP17), which have been associated with oocyst reduction rates of 82.75%, 63.4%, 61.43%, and 64.29%, respectively (15–18). Thus, *Et*ROP21 might be a good antigen candidate for the development of vaccines against chicken coccidiosis, and its functions in the invasion, survival, and growth of *E. tenella* need further investigation.

## 5 Conclusion

Our analysis revealed that the actual length of the *Et*ROP21 gene fragment expressed by *E. tenella* sporozoite is shorter than the putative whole *Et*ROP21 gene recorded in the database of National Center for Biotechnology Information (NCBI). The recombinant *Et*ROP21 (r*Et*ROP21) expressed in *E. coli* had a molecular weight of approximately 50 kDa. Two specific protein bands, about 43 KDa and 95 KDa in size, were detected in the whole sporozoite proteins using the r*Et*ROP21-immunized chicken serum in the Western blot. The larger protein (95 KDa) was speculated to be the dimer form of the *Et*ROP21 protein. Immunofluorescence and immunoelectron assays showed that in *E. tenella* sporozoites, the *Et*ROP21 protein was localized at the bulb region of rhoptries and that it was predominantly distributed at two terminals and perinuclear regions. Furthermore, r*Et*ROP21 could induce specific humoral and cellular immune responses, offer robust immunoprotection against *E. tenella* infection, and suppress oocyst levels by 67.42%. These findings highlight *Et*ROP21 as a potential candidate for the development of vaccines against coccidiosis in chickens. However, its functions in the invasion, survival, and virulence of *E. tenella* require further study.

## Data availability statement

The original contributions presented in the study are included in the article/**Supplementary Material**. Further inquiries can be directed to the corresponding authors.

## Ethics statement

The animal study was approved by Institutional animal care committee of the Zhejiang Academy of Agricultural Sciences. The study was conducted in accordance with the local legislation and institutional requirements.

## Author contributions

T-yS: Funding acquisition, Project administration, Conceptualization, Data curation, Writing – original draft. S-hZ: Writing – original draft, Methodology, Software. Y-rK: Formal Analysis, Investigation, Writing – review & editing. YF: Conceptualization, Methodology, Resources, Validation, Writing – review & editing. YL: Data curation, Resources, Software, Writing – review & editing. W-cY: Conceptualization, Methodology, Supervision, Validation, Writing – review & editing. Y-xZ: Conceptualization, Data curation, Formal Analysis, Writing – review & editing. LZ: Investigation, Methodology, Visualization, Writing – review & editing. L-lH: Formal Analysis, Methodology, Software, Writing – review & editing. H-cS: Funding acquisition, Methodology, Project administration, Writing – review & editing.

## Supplementary material

Supplementary Figure 1 Construction of the prokaryotic expression plasmid, pET32a-*Et*ROP21. M1 & M2: DNA markers. **(A)** The *Et*ROP21 fragment was amplified using PCR. Lane 1: the product of the PCR analysis of the actin gene in *E. tenella*. Lane 2: the product of the PCR using the primer pair, rop21-F11 and rop21-R. **(B)** The expression plasmid, pET32a-*Et*ROP21, was identified via double digestion. Lane 1: the product of the recombinant plasmid, pET32a-*Et*ROP21, after digestion with the restriction enzymes, *Kpn*I and *Xho*I.
