# Supplementary figures and images for: A rhoptry protein, localizing in the bulb region of rhoptries, could induce protective immunity against Eimeria tenella infection
Source: Front Immunol. 2023 Dec 4;14:1277955. doi: 10.3389/fimmu.2023.1277955 (PMC10725939; doi:10.3389/fimmu.2023.1277955)

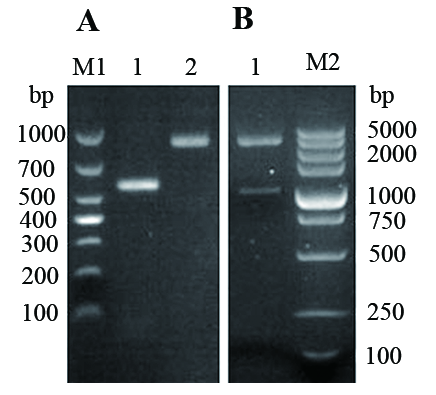

Supplement: Supplementary Figure 1 — Construction of the prokaryotic expression plasmid, pET32a-EtROP21. M1 & M2: DNA markers. (A) The EtROP21 fragment was amplified using PCR. Lane 1: the product of the PCR analysis of the actin gene in E. tenella. Lane 2: the product of the PCR using the primer pair, rop21-F11 and rop21-R. (B) The expression plasmid, pET32a-EtROP21, was identified via double digestion. Lane 1: the product of the recombinant plasmid, pET32a-EtROP21, after digestion with the restriction enzymes, KpnI and XhoI. [file Image_1.tif]
